# Ocular Trauma: An Overview

**DOI:** 10.5812/atr.21639

**Published:** 2014-06-29

**Authors:** Dawood Aghadoost

**Affiliations:** 1 Trauma Research Center, Kashan University of Medical Sciences, Kashan, IR Iran

**Keywords:** WHO, Blindness

Blindness, especially when bilateral, is a serious public health problem that affects a person's quality of life and imposes major socioeconomic and psychological impacts on patients and their relatives. The number of blind peoples increases to 76 million in year 2020 if no active preventive measure is installed. Incidence of blindness varies in different communities ranging from 0.2-1.0 percent by WHO sub region (1).

The most common cause of unilateral blindness in pediatric age groups, especially in developing countries, is ocular trauma. It is simply preventable by the supervision of the parents and baby caregivers (2).

The epidemiology of eye injuries varies in different parts of the world and different age groups and depends on many factors including life style, socioeconomic status, traffic state, sport and creative activities and type of registration and recording of data (3). About half a million people in the world are blind as a result of eye injuries. About 30-40% of monocular blindness is due to ocular trauma (2, 3).

Ocular injuries, even minor types, may result in significant economic burdens to families and countries due to time lost from work, or school and family care giving, expensive hospitalization, specialist visit and treatment, prolonged follow-up and visual rehabilitation (4). Estimation of direct and indirect costs of ocular trauma is difficult because it needs accurate data which is not accessible without definite strategies and planning.

In many descriptive studies in the world, the major risk factors and epidemiologic features are age, gender, socioeconomic status and life styles. Review of literature from 1992–2013 showed no significant changes in pattern, etiology and location of occurrence of eye injuries (5-10). High rates of eye trauma occur in young males (age 18-25 years), and this is related to work, sport, assaults and traffic (2-4). Less common causes of eye injuries are BB gun shot which usually leads to severe visual impairment despite modern surgical techniques (2, 4), war- related ocular injuries (8) and fireworks in children (6). Contact lens- induced keratitis and decreased vision is increasing due to wide–spread use of contact lenses (7).

About 38-52% of all cases presenting to ophthalmic emergency rooms are ocular trauma and 0.9-1.8% of them need to be admitted due to severe trauma (4).

Although most cases of traumatic eye injuries are preventable, current preventable strategies for them need more effective implementation. Today, preventive measures and protective eye wears have reached to work places, sport and leisure facilities; however, they are neither readily available for use, nor comfortable during related activities.

It is hopefully supposed that training about hazards associated with specific activities, facilitating the availability of eye wears, accident prevention and training baby care givers for prevention of children trauma may prevent or decrease mortality and morbidity of ocular injuries.

A standardized international design for recording of eye injuries seems to be mandatory to permit accurate planning for the prevention and management of this disastrous incident.
